# Screen Printed Antennas on Fiber-Based Substrates for Sustainable HF RFID Assisted E-Fulfilment Smart Packaging

**DOI:** 10.3390/ma14195500

**Published:** 2021-09-23

**Authors:** Jarne Machiels, Raf Appeltans, Dieter Klaus Bauer, Elien Segers, Zander Henckens, Wouter Van Rompaey, Dimitri Adons, Roos Peeters, Marie Geiβler, Katrin Kuehnoel, Lydia Tempel, Thomas Weissbach, Arved Carl Hübler, Akash Verma, Eleonora Ferraris, Wim Deferme, Mieke Buntinx

**Affiliations:** 1 Materials and Packaging Research & Services, Institute for Materials Research (IMO-IMOMEC), Hasselt University, Wetenschapspark 27, B-3590 Diepenbeek, Belgium; jmachiels@tacon.nl (J.M.); elien.segers@uhasselt.be (E.S.); zander.henckens@uhasselt.be (Z.H.); wouter.vanrompaey@uhasselt.be (W.V.R.); dimitri.adons@uhasselt.be (D.A.); roos.peeters@uhasselt.be (R.P.); 2 Functional Materials Engineering, Institute for Materials Research (IMO), Hasselt University, Wetenschapspark 1, B-3590 Diepenbeek, Belgium; wim.deferme@uhasselt.be; 3 IMEC vzw, Division IMOMEC, Wetenschapspark 1, B-3590 Diepenbeek, Belgium; 4 IMEC vzw, Kapeldreef 75, B-3001 Leuven, Belgium; raf.appeltans@imec.be; 5 Fraunhofer Institute for Process Engineering and Packaging IVV, Giggenhauser Str. 35, 85354 Freising, Germany; klaus.dieter.bauer@ivv.fraunhofer.de; 6 Papiertechnische Stiftung (PTS), Pirnaer Straβe 37, 01809 Heidenau, Germany; marie.geissler@ptspaper.de (M.G.); Katrin.Kuehnoel@ptspaper.de (K.K.); lydia.tempel@ptspaper.de (L.T.); 7 Institute for Print and Media Technology, Technische Universität Chemnitz, Reichenhainer Str. 70, 09126 Chemnitz, Germany; thomas.weissbach@mb.tu-chemnitz.de (T.W.); arved.huebler@mb.tu-chemnitz.de (A.C.H.); 8 Manufacturing Processes and Systems, Department of Mechanical Engineering, KU Leuven, J. D. Nayerlaan 5, B-2860 Sint-Katelijne Waver, Belgium; akash.verma@kuleuven.be (A.V.); eleonora.ferraris@kuleuven.be (E.F.)

**Keywords:** screen printing, radio frequency identification (RFID), antenna, paper substrate, recyclability, intelligent packaging, e-fulfilment, transport simulation

## Abstract

Intelligent packaging is an emerging technology, aiming to improve the standard communication function of packaging. Radio frequency identification (RFID) assisted smart packaging is of high interest, but the uptake is limited as the market needs cost-efficient and sustainable applications. The integration of screen printed antennas and RFID chips as smart labels in reusable cardboard packaging could offer a solution. Although paper is an interesting and recyclable material, printing on this substrate is challenging as the ink conductivity is highly influenced by the paper properties. In this study, the best paper/functional silver ink combinations were first selected out of 76 paper substrates based on the paper surface roughness, air permeance, sheet resistance and SEM characterization. Next, a flexible high frequency RFID chip (13.56 MHz) was connected on top of screen printed antennas with a conductive adhesive. Functional RFID labels were integrated in cardboard packaging and its potential application as reusable smart box for third party logistics was tested. In parallel, a web-based software application mimicking its functional abilities in the logistic cycle was developed. This multidisciplinary approach to developing an easy-scalable screen printed antenna and RFID-assisted smart packaging application is a good example for future implementation of hybrid electronics in sustainable smart packaging.

## 1. Introduction

Packaging is considered to protect a product from physical damage and to ensure its quality during transportation, storage and end use. The major functionalities of conventional packaging are containment, machinability, utility and convenience, protection and communication and information. Intelligent packaging is an emerging technology, which aims to improve the standard communication function by monitoring, detecting, sensing, recording, tracking and/or communicating [1,2]. The ability to measure and/or communicate a specific property of the packaged product, the internal atmosphere, or the external environment provides enormous potential regarding safety, quality and traceability, as well as its convenience for consumer interaction [1,3]. Indicators provide visual or semi-quantitative information about changes in quality or surrounding conditions (e.g., pH, freshness, temperature), while sensors are used for detection, quantification and quality evaluation of physical or chemical characteristics of the packaged goods [2,4]. QR codes, barcodes or radio frequency identification (RFID) tags are widely used for counterfeit protection, storage and traceability purposes [2,5,6]. Information obtained from an intelligent system can be communicated to stakeholders in the supply chain or to consumers, or can eventually trigger active packaging functions. Active packaging goes beyond the inert passive containment and protection of the packaged product by specifically interacting with the product using scavengers, emitters or adapters [7,8,9].

The domain of intelligent packaging is a dynamic and high-growing market influenced by varying lifestyles, the necessity of manufacturers to create products with enhanced shelf life, and stringent management regulations on safety standards [10,11,12]. The use of data carriers such as 2D barcodes, RFID and near field communication (NFC) is highly increasing in interest [3,13]. This study focuses on RFID-assisted packaging because of its unique identification (UID) without human intervention and promising advantages in supply chain management [14,15]. RFID systems exploit electromagnetic waves to transfer data wirelessly, in particular used for automatic identification, data capture and tracking purposes. The two-way data transferring method consists of a transponder or tag (located on the object to be identified) and an interrogator or reader to communicate between the tag and the user software. These tags are classified by their working frequency, directly related to the reading distance [16]: low frequency (125 kHz; <1 m), high frequency (HF) (13.56 MHz; 0.9–2.5 m) and ultrahigh frequency (UHF) (400–900 MHz; up to 10 m). Active tags always use their own energy supply (e.g., battery, solar cell) to provide voltage to the chip, while passive systems directly harvest power from the radio-electric energy of the reader. Semi-active tags are connected to an external power source, but only use that extra power for high power consuming tasks [17,18].

To achieve low-cost RFID assisted packaging, the development of RFID systems that allow communication without an expensive external power supply is required. Passive RFID tags–consisting of a microchip and an antenna that receives modulated and decrypted signals from the chip to send a response signal back to the interrogator [19]–are traditionally manufactured using subtractive processes like photolithography, coil winding and etching techniques. Although these methods are reliable and accurate, disadvantages such as material waste, process complexity and high cost [20] have prompted us to look for alternatives. Additive printing processes for electronic applications [21,22], such as screen printing, flexography, gravure printing, inkjet printing, etc., have been investigated because of their cost efficient, simple and fast manufacturing processes [23,24,25]. The future scenario aims at enabling production and integration of functional electronic components (at ambient conditions) directly in printing or conversion lines. Though the proper selection of the RFID antenna printing technique is a critical factor in this process, because it directly affects the ink requirements, substrate selection and printing resolution, ‘Printed Electronics’ will be a megatrend in food and pharmaceutical packaging because it is an enabling technology and accelerator for other megatrends such as the Internet of Things (IoT), consumer interaction via mobile phones, traceability and safety during online retail by surveillance of storage and transport conditions, etc. [26,27].

Currently, screen printing is one of the dominant printing technologies of functional materials on different flexible and low-cost substrates such as plastic (e.g., polyethylene terephthalate, polyimide…), textile and paper [28]. Using paper as a printing substrate has ecological and economic benefits because it is made from renewable resources, it is recyclable and substantially cheaper [10]. Whereas plastics generally feature smooth, non-absorbent surfaces, paper properties can vary to a large extent depending on the composition, structure and environmental conditions (e.g., relative humidity) [29,30]. Although fiber-based substrates are widely used in the conventional printing industry, the field of printed electronics on paper or cardboard substrates is rather limited [29,31]. The observed challenges towards printability and electrical performance are related to the surface roughness, absorption capacity, barrier properties, thermal stability and surface energy of fiber-based substrates [29,32,33]. Despite these hurdles, Xie et al. have shown that ordinary low-cost paper is suitable for inkjet printed smart packaging applications with optimized properties of conductivity [34] and Pereira et al. have demonstrated the potential of screen printing on paper substrates to develop NFC tags [35].

Printed RFID antennas have already been investigated on different substrates [36]. Baumbaumer et al. demonstrated that screen printing and stencil printing are suitable for antenna fabrication on polyethylene napthalate (PEN) [37]. UHF RFID antennas are most common because the tunability at their working frequency band is not a challenging feature. In [38,39,40], inkjet printing technology is used to develop UHF RFID antennas by deposition of silver nanoparticle conductive ink on photo paper. Shin et al. [41] and Jaakola et al. [42] reported the use of screen printing for the development of UHF RFID dipole antennas on polyethylene terephthalate (PET) and polyimide (Kapton) respectively, while other studies investigated screen printed UHF RFID tags on paper [43,44]. In the work of Fernández-Salmerón et al., a UHF RFID dipole antenna was directly screen printed on a cardboard package [45]. In contrast to UHF RFID antennas printed on fiber-based substrates, the number of printed HF RFID antennas on paper is still limited. Polymeric substrates (e.g., PET film) [46] and glossy photo paper with a planarization coating [47], have shown the ability to screen print HF RFID antennas. However, the operating frequency at 13.56 MHz complicates the development of a printed HF antenna, because its range in electrical and RF performance is extremely limited, especially in combination with varying paper substrate characteristics.

**Figure 1:**
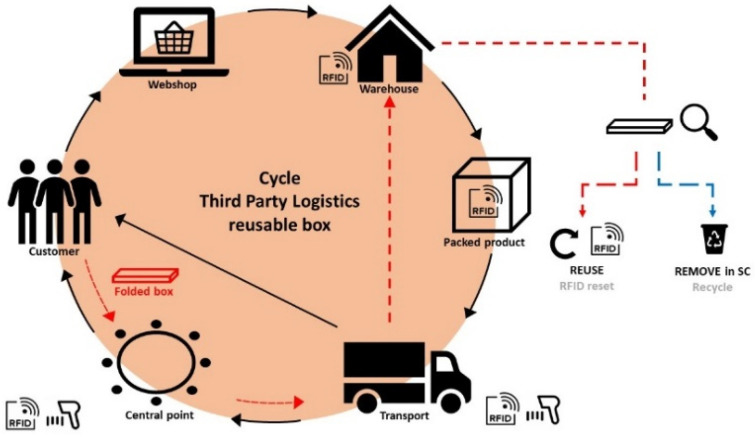
Cycle of reusable packaging within third-party logistics.

The objective of this work is to design and develop a sustainable HF RFID assisted e-fulfilment package with multiple scanning interactions within third-party logistics (3PL). The concept is clarified in Figure 1.

E-fulfilment covers the essential arrangements for stakeholders to sell their products or services to the customer online [48]. The operational logistics, such as transportation, warehousing, packaging and even tracking, are generally outsourced to a third service company (3PL). The rising demand for circular packaging that guarantees product protection and minimalizes waste, challenges logistic companies to innovate [10,49]. They are stimulated to reuse the secondary packaging, which is used to protect and bundle primary packages. Unfortunately, the current trippage number (times the package is reused as part of its lifecycle) is mostly zero [50].

This study starts from 76 fiber-based substrates and 2 conductive silver inks, which are provided by stakeholders in the smart packaging supply chain. First, the influence of paper properties–such as surface roughness and air permeance–on the sheet resistance of screen printed structures is investigated. Suitable substrates for printed electronics are selected based on their printability and ink compatibility performance. Next, conductive silver ink, fiber-based substrate, print design and print parameters are properly combined to develop an easy-scalable screen printed HF RFID antenna, which is connected to a flexible thin-film microchip and validated. In addition, the recyclability of the screen printed antennas is investigated according to the PTS-RH: 021/97 standard. Finally, a tripartite foldable cardboard package with integrated HF RFID tag is designed, developed and validated to obtain a functional, reliable and sustainable HF RFID assisted e-fulfilment packaging.

## 2. Materials and Methods

### 2.1. Materials

76 different fiber-based substrates such as (un)coated paper and cardboard were kindly provided by industrial partners. Considering the requirement for low sheet resistance (<50 mΩ/sq) to print HF RFID antennas, two conductive nano silver inks were selected: ‘Orgacon SI-P2000’ from Agfa-Gevaert (Mortsel, Belgium) and ‘Loctite ECI 1011’ from Henkel (Düsseldorf, Germany). Both inks are highly conductive with a sheet resistance below 5 mΩ/sq, as measured on a PET foil after an oven sintering at 150 °C for 10 min. A 12-bit HF RFID IGZO-TFT (indium-gallium-zinc-oxide thin-film transistor) microchip, designed and developed by IMEC (Leuven, Belgium), was connected on top of the screen printed antennas with the electrically conductive adhesive ‘IQ-BOND 5402-CE’ from Roartis (Genk, Belgium). Double-walled corrugated cardboard (580 g/m²) with EE flute type (EE606 from Cartonneries De Gondardennes SA, Wardrecques, France) was used to develop a foldable cardboard package with outer dimensions of 425 × 315 × 195 mm^3^. Strapping bands were selected as an adhesive-free alternative to close or seal the tripartite box. Finally, 35 µm transfer tape (6035 from Tape Converters Holland BV, Weert, The Netherlands) was used to adhere the HF RFID tag onto the cardboard package.

### 2.2. Characterization and Deposition of Ink on Fiber-Based Substrates

The surface roughness value Ra (nm) of the fiber-based substrates was determined using a DektakXT Stylus Profiler (Bruker, Billerica, MA, USA) by 6 single line measurements (1.8 mm). The air permeance (mL/min) was characterized using the L&W Bendtsen Tester, type SE 114 (Munich, Germany) (ISO 5636-3) by 10 single measurements. The air flow rate, measured in mL/min, is an alternative measure of the substrate’s air permeability per unit thickness which is indirectly related to its absorption behavior (i.e., porosity). The data do not offer quantitative information on the pore size or geometry, but they can be used to compare qualitatively the porosity of the paper substrates [30,51]. The surface topography (Ra and Rz) of selected paper substrates was also evaluated using the Focus Variation method of an Alicona Infinite Focus Microscope G3.

Flat-bed screen printing (ISIMAT 1000 PE, Ellwangen, Germany) with a 40 × 40 meshed PET screen (SEFAR PET 140/355-31, 40 threads per cm) was carried out to investigate the relationship between the surface roughness and air permeance of the substrate and the electrical properties of the printed inks. A polyurethane squeegee with a 70 to 75 Shore hardness and sharp edge was applied to deposit the inks (75 durometer straight edge squeegee with a width of 100 mm). The distance between the screen and substrate during printing is 5 mm with an angle of 16° and a print speed of 100 mm/s, at a squeegee pressure of 2.5 bar resulting in a force of 66 Newton. Small squares (10 × 10 mm) of both conductive nano silver inks were deposited on all paper and cardboard substrates and the printed samples were thermally cured for 10 min at 150 °C in a ventilated oven. As a post-curing characterization, the sheet resistance (mΩ/sq) of the silver layers was measured by a home-built Van der Pauw measurement system. By applying this four-point probe method, the voltage and current electrodes are separated to eliminate the contact resistance [52].

SEM analysis of a selection of samples has been performed with a scanning electron microscope (JSM-7200F, JEOL Ltd., Tokyo, Japan). In order to reduce electrical charging of the non-conducting samples by the electron beam, all specimens had been sputter-coated with a thin gold layer (a few nanometers) prior to observation. Images have been obtained using the secondary electron detector (LED mode). A low acceleration voltage of 1 kV has been chosen to give a good reproduction of the topography and also to reduce sample damage at high resolution (magnification).

Viscosity measurements were performed at 25 °C on a MCR301 (Anton Paar, Austria) rotational rheometer in a plate-cone configuration with a diameter of 49.98 mm diameter. 1.008° cone angle and 97 µm cone truncation. The samples were equilibrated for at least 3 min before starting the experiment. The shear rate was increased by 41 steps on a logarithmic scale from 0.01 1/s to 1000 1/s. At each step, the shear rate was kept constant for 15 s.

### 2.3. Recyclability Testing

Recyclability trials, according to the PTS-RH 021:2012 method, were performed on three unprinted substrates from the ultimate substrate selection and the functional printed RFID antenna. To assess these materials, they were pulped for 10 min with a standard disintegrator. The disintegration behavior was determined using a Brecht-Holl-Fractionator (0.7 mm hole) (PTS-RH 021:2012) [53]. The total stock was then screened with a slot plate (0.15 mm slot, Haindl fractionator) and the accepted pulp was used to form hand sheets for an inspection on tacky and visual impurities.

### 2.4. Antenna Design and Integration

**Figure 2:**
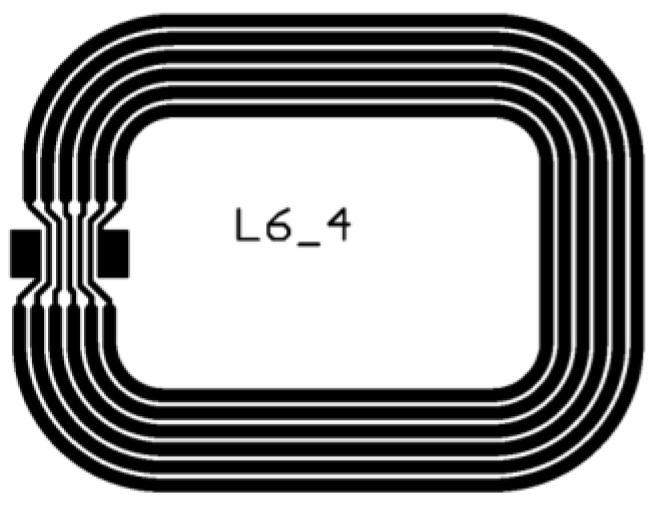
High frequency RFID antenna design (L6_4), designed by IMEC as described by [54].

The investigated antenna design is a square-shaped, post-it sized loop antenna (Figure 2) as described by Machiels and Verma et al. [54]. It resonates at a 13.56 MHz base carrier frequency and should meet the requirements of series resistance (20–25 Ω) and inductance (2.90–3.00 µH).

Flat-bed screen printing (mesh: 140 T/cm; 31 µm diameter PET fiber) with both conductive nano silver inks was carried out to deposit antennas onto the selected paper substrate ‘Magno Satin’ (135 g/cm²) from Sappi Europe (Brussels, Belgium). The same polyurethane squeegee (2.2) was applied to deposit the inks. As a post-printing step, the antennas were thermally cured for 10 min at 150 °C in a ventilated oven. The printed RFID antennas were initially characterized by basic electrical measurements for their series resistance and inductance with a HM8118 LCR meter (Rohde & Schwarz, Cologne, Germany) at 1 kHz and 1 V. In addition, the RFID functionality of the antenna is tested in combination with the microchip by extracting the tag’s code with a specific reader device. The IGZO-TFT microchip itself contains an analogue to digital convertor (ADC, up to 6-bit) and wireless ICs (Integrated Circuit) with NFC ISO 14443-A barcode and capacitive identification capabilities. For calculating capabilities, it has a plastic microprocessor and a laser-programmable memory (LPROM). Integration was done by QUAD Industries (Sint-Niklaas, Belgium).

### 2.5. Design and Functionality Testing of a Sustainable E-Fulfilment Package

**Figure 3:**
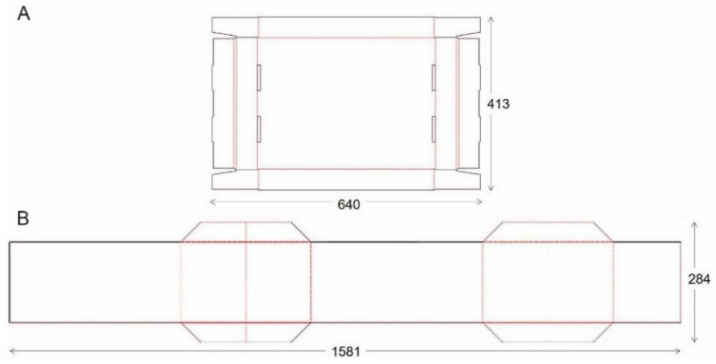
Technical drawing of ‘bottom/lid’ (**A**) and ‘mantle’ of the tripartite foldable box (**B**).

A cutting table (Kongsberg XL22 from Esko, Gent, Belgium) was used to cut the cardboard parts according to the technical drawing (Figure 3) of the tripartite foldable box (42.5 × 31.5 × 19.5 cm^3^). Both the bottom and the lid were customized by perforation lines and protuberances to improve the box’ rigid structure. The HF RFID tag can be placed in the box’ lid between two corrugated cardboard sides for protection and visibility during transport.

The cardboard used to assemble the box was initially conditioned at 50 ± 2% relative humidity (RH) and 23 ± 1 °C for 48 h (ISO 187 standard) before functionality testing was performed. The cardboard’s single sheet thickness (mm) was determined using a L&W Micrometer, type SE 050 (Munich, Germany) in accordance with the ISO 3034 standard. The amount of energy (J) used to puncture the cardboard was determined as verification to withstand external forces (e.g., during transport, forklift truck, etc.) using an Analogic Puncture Tester type PM-01 from Metrotec (Lezo, Spain) in accordance with the ISO 3036 standard. The strength of the cardboard waves, i.e., the edge crush resistance ECT (N) was measured to conform to the ISO 3037 standard using a L&W Crush Tester, type SE 048 (Munich, Germany). The compressive strength (N) of the complete box was measured in the box compression test (BCT) using an MTS 10/M compression device (Eden Prairie, MI, USA) in accordance with the ISO 12048 standard. Finally, transport simulation experiments (ASTM D4169-16) were carried out on filled boxes to investigate the performance of shipping containers and systems by testing their ability to resist the distribution environment. The test cycle consisted of different sequences, based on distribution cycle 13 (DC 13) (air (intercity) and motor freight (local single package up to 61.8 kg)), resulting in the following specially defined distribution cycle, user specified (DC 2), assurance level II (moderate risk) and box mass lower than 9.1 kg:

- Manual handling (schedule A): drop test from 381 mm height (ASTM D5276) using the Lansmont PDT 80 Precision Drop Tester (Monterey, CA, USA);
- Vehicle stacking (schedule C): calculation of maximum package’s load without collapsing using the TechLab Systems VAL100 compression device (Lezo, Spain);
- Loose-load vibration (schedule F): determination whether the package can withstand repetitive shocks using the Lansmont Vibration Test System 10000 (Monterey, CA, USA);
- Vehicle vibration (schedule E): simulation of 1 h truck-and 2 h air transport using the Lansmont Vibration Test System 10000;
- Manual handling (schedule A): drop test from 381 mm height, except bottom side from 762 mm height, using the Lansmont PDT 80 Precision Drop Tester.

## 3. Results and Discussion

### 3.1. Selection of Fiber-Based Substrates Based on Printability and Ink Compatibility

The electrical performance of printed electronics on fiber-based substrates highly depends on inherent paper characteristics such as surface roughness, air permeance (i.e., absorption behavior), surface energy, thermal stability (i.e., resistance to heat curing), bending stiffness etc. [29,30,54]. To select the most suitable fiber-based substrates out of 76 commercial papers that were considered to be of interest for printed electronics by industrial partners, square-shaped silver prints were screen printed on the diverse set of (un)coated paper and cardboard samples. Next, the sheet resistance of the silver prints was measured and the relationship between sheet resistance and the substrate properties such as surface roughness and air permeance was determined. The surface roughness of a paper substrate denotes the deviation of the surface in its normal direction. Papers with higher surface roughness have a higher ratio of surface area to volume, and thus, they can store more ink [55]. Air permeance describes the permeability over the length of the flow path which is indirectly related to the porosity as described by Ihalainen et al. [30]. Sheet resistance is used here only to compare appropriate fiber-based substrates. For the final application of antennas however, the resistance value, together with the inductance value, are more relevant parameters.

**Figure 4:**
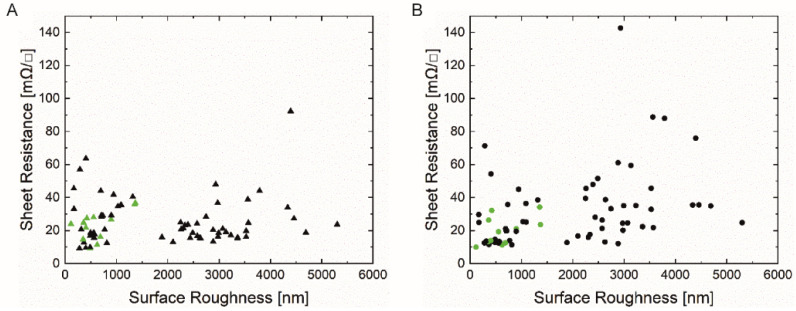
Sheet Resistance as function of Surface Roughness of 5 ‘Orgacon SI-P2000’ (▲, **A**) and ‘Loctite ECI 1011’ square prints (●, **B**). Green symbols indicate the 13 paper samples that were selected for further investigation.

The direct-contact surface profilometry technique tracked the substrates’ profile to determine the average surface roughness value Ra (nm). Figure 4A,B show the measured sheet resistance (mΩ/sq) of both square-shaped silver prints (‘Orgacon SI-P2000’ (A), ‘Loctite ECI 1011’ (B)) on all tested substrates as function of their measured surface roughness. All measurements were performed under the same conditions (room temperature and general lab relative humidity).

**Figure 5:**
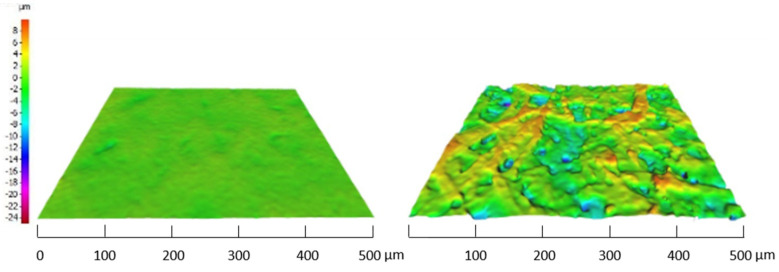
3D-visualisation of the surface topography of two different paper types. The left image shows a smooth paper sample (Algro Baress) (Ra = 0.29 μm), with relatively low peak-to-peak value (Rz = 5.02 μm). The right image shows a sample (Schutpaper–Acryl/Latex impregnated) with a higher mean surface roughness (Ra = 2.16 µm) and higher peak-to-peak value (Rz = 22.16 µm). The Ra values are in the same range as the DektakXT Stylus Profiler measurements.

Both graphs indicate that a wide range of surface roughnesses are included, varying from 114 ± 15 nm for the smoothest substrate (p_e:smart paper type 2, Felix Schoeller) to 5301 ± 792 nm for the roughest substrate (M300, VPK Packaging). The corresponding sheet resistance value is unique for each substrate, depending on the used ink and paper characteristics (all data are shown in the Supplementary Table S1). According to Figure 4, there is no correlation between the paper surface roughness and the sheet resistance values. In general, rough surfaces can cause discontinuation of the print and thus decrease the electrical conductivity. In conjunction with the mean roughness values (Ra), as provided in Figure 4, the peak-to-peak values (Rz) could give better insight into the surface characteristics. Figure 5 shows a 3D-visualisation of the surface topography of two paper substrates; one with low Ra values (Algro Baress, left) and one with higher Ra values (Schutpapier, right). As can be seen, the Rz values also follow the same trend. These higher peak-to-peak values can cause discontinuation of the print. For this reason, it is still recommended to use smooth surfaces in the field of printed electronics on paper substrates [29,30]. In this study, the thickness of the screen printed layer (ranging between 6 to 7 µm) (data not shown) exceeds the surface roughness of all paper substrates and for that reason, low sheet resistance values might have been observed. The roughness of the printed films is not mentioned as we have used the measured total thickness and sheet resistance values in this study to select suitable paper substrates for further experiments.

**Figure 6:**
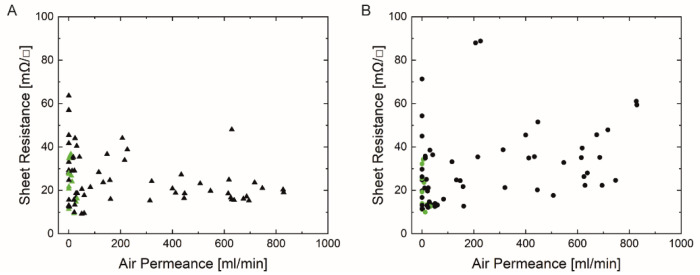
Sheet Resistance as function of Air Permeance of ‘Orgacon SI-P2000’ (▲, **A**) and ‘Loctite ECI 1011’ square prints (●, **B**). Green symbols indicate the 13 paper samples that were selected for further investigation.

Figure 6A,B present the sheet resistance values of the square-shaped silver prints as a function of the air permeance (mL/min) of the paper substrates. The latter monitors the air flow across the substrate, and it could be related to its porosity and absorption behavior [30]. In general, it is desired to use non-porous over porous substrates to prevent excessive absorption of the functional ink and to achieve homogenous conductive layers on top of the substrate [29,32]. On the other hand, a minimum amount of ink penetration is required for adhesion, and thus stability and durability of the silver prints. The measured air permeance values varied over a wide range; from impermeable (i.e., coated substrates) to 829 ± 44 mL/min (UPM Poste 120). No correlation was observed between the air permeance of the substrates and the sheet resistance for both inks. However, as shown in Figure 6, the sheet resistance of many high permeable samples printed with ‘Loctite’ ink was higher compared to the ‘Orgacon’ ink.

**Figure 7:**
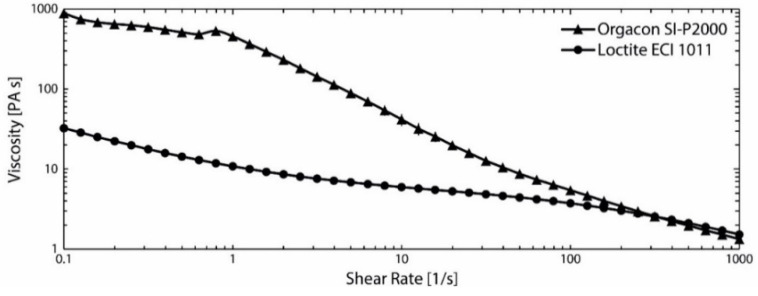
Viscosity as function of Shear Rate of ‘Orgacon SI-P2000’ (▲) and ‘Loctite ECI 1011’ (●). Each value is the average of three measurements. The standard deviation is below 5%.

Since viscous screen printing pastes, which do not penetrate fiber-based substrates that extensively (as compared to low-viscous inkjet inks) were used, these results suggest that screen printing is a promising deposition technique for printed electronics on paper substrates. The fact that the observed sheet resistance values of ‘Orgacon SI-P2000’ prints are generally lower than of ‘Loctite ECI 1011’ prints, might be a consequence of the higher viscosity of the Orgacon ink versus the Loctite ink at low shear rates (see Figure 7), and on the lower volume resistivity of the ‘Orgacon SI-P2000’ ink (3 mOhm/sq/25 μm) versus the Loctite ink (5 mOhm/sq/25 μm). Especially at higher air permeance, the penetration into the paper is more evident for the lower viscous ink resulting in a worse covering of the top structure and thus a higher sheet resistance. The higher viscous ink will not penetrate that easily, resulting in a more homogeneous coverage on top of the paper, and thus in lower sheet resistances. In addition, the deposited layer’s thickness can exceed the ‘disturbing’ surface roughness of paper. The fact that some impermeable substrates exhibit higher sheet resistance values is probably caused by other paper characteristics (e.g., surface roughness, wetting behavior). Tobjörk and Österbacka [29] and Ihalainen et al. [30] have suggested that the best types of paper substrates for printed electronics yield low values for both surface roughness and air permeance so that the ink is uniformly deposited without excessive absorption.

**Figure 8:**
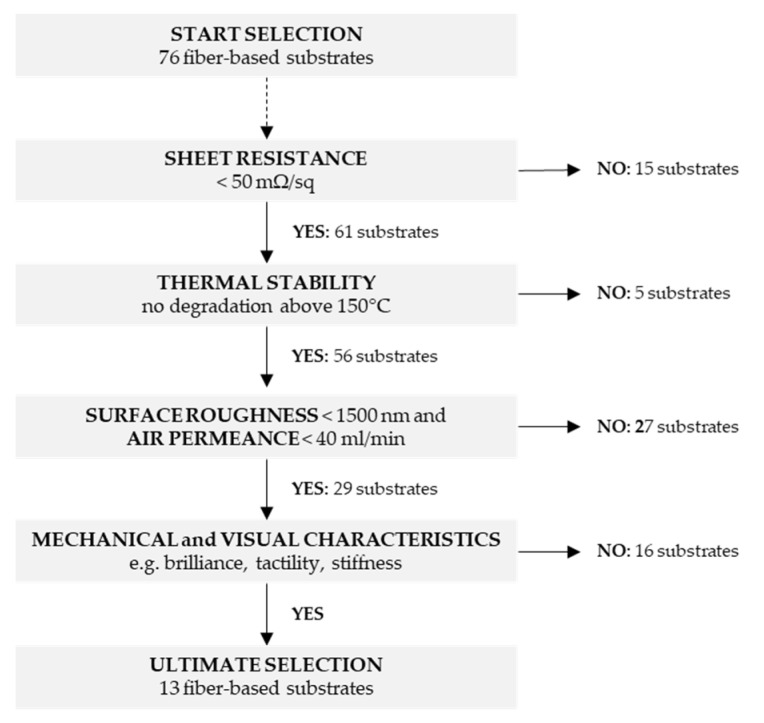
Elimination criteria with threshold values used to downscale the number of fiber-based test substrates towards a selection of substrates with suitable properties for printed electronics.

Although no clear correlation was found between the paper characteristics (surface roughness and air permeance) and sheet resistance values (as measured using the Van der Pauw method), the experimental data allowed to distinguish proper ink-substrate interactions which are a balance between wetting, absorption and solidification between both. The next step of this work involved the final selection out of 76 tested substrates. Suitable substrates are defined to feature low sheet resistance (<50 mΩ/sq), no degradation after exposure to temperatures above 150 °C (during the curing process), low surface roughness (<1500 nm) and low air permeance (<40 mL/min). The low threshold value for sheet resistance is required for HF RFID antenna printing whereas the threshold values for surface roughness and air permeance were introduced as result of the qualitative/qualitative observations in the printability experiments. By using these criteria, we assume that deposition of undesired pinholes or non-homogenous prints can be avoided. The elimination criteria are shown in Figure 8. The introduction of the threshold values for sheet resistance, thermal stability (i.e., heat deformation), surface roughness and air permeance reduced the number of suitable substrates to 29 (out of 76). Finally, the other 16 substrates were eliminated based on mechanical and visual paper characteristics (e.g., brilliance, tactility, stiffness).

Table 1 shows the ultimate selection of fiber-based substrates that were further used in this study because they were considered to be the best compatible with printed electronics applications.

**Figure 9:**
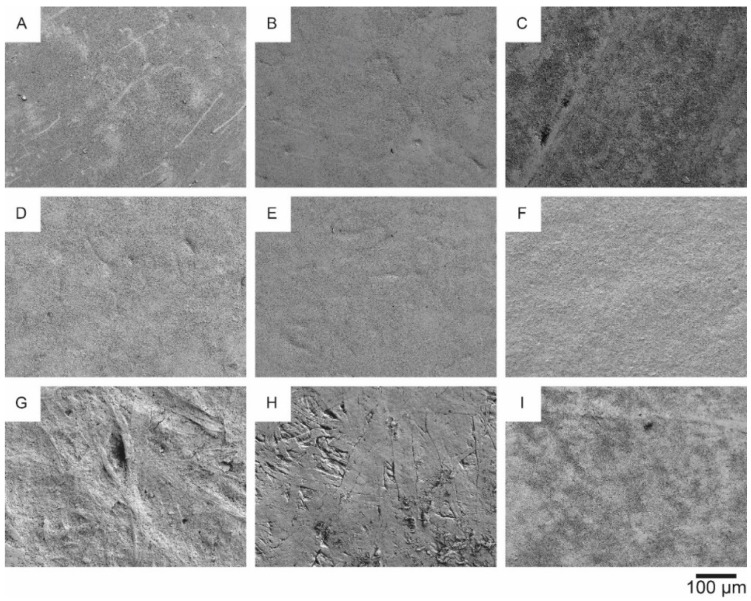
SEM images at a magnification factor of 200 of the following substrates: p_e:smart paper type 2 (**A**), Parade Label A (**B**), Incada Exel (**C**), Koehler Type E (**D**), Algro Baress (**E**), Magno Satin (**F**), Silvaboard (**G**), PG90 (**H**), UPM Finesse Premium Silk H 90 (**I**).

The smoothest substrates listed in Table 1 have also been analyzed by SEM. Figure 9 presents the images of the respective surfaces with a magnification factor of 200. It is obvious that all of these fiber-based substrates have been coated by the producers, except the PG90 from Grünperga.

**Figure 10:**
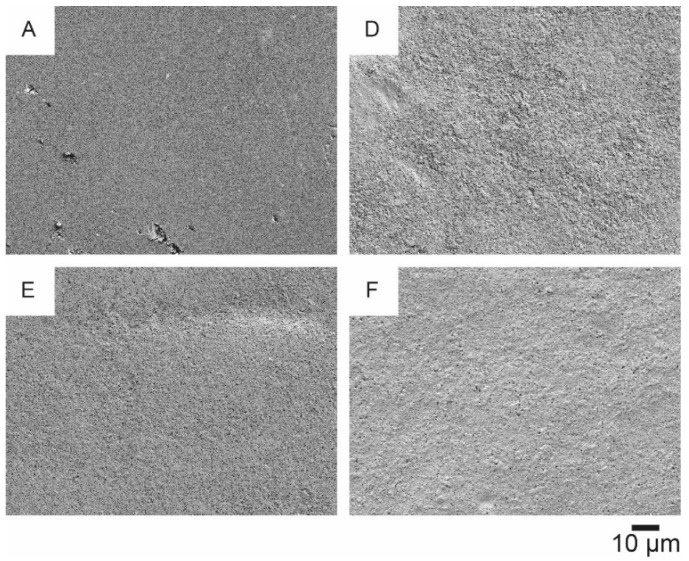
SEM images at a magnification factor of 1000 of the following substrates: p_e:smart paper type 2 (A), Koehler Type E (D), Algro Baress (E), Magno Satin (F).

**Figure 11:**
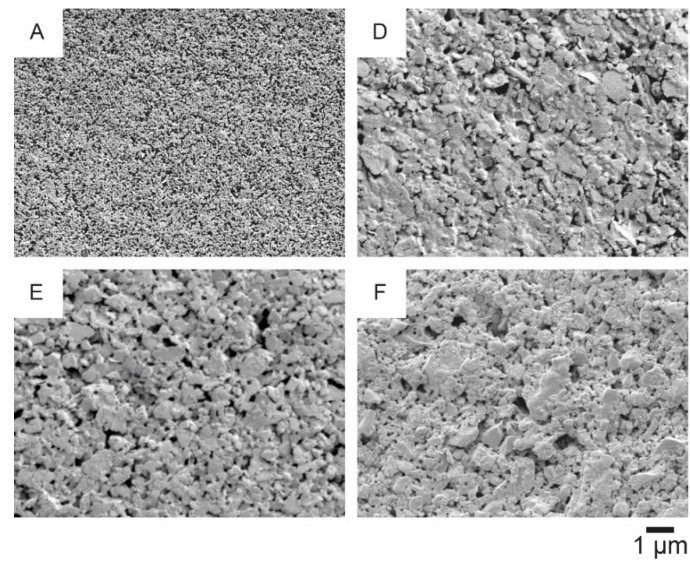
SEM images at a magnification factor of 10,000 of the following substrates: p_e:smart paper type 2 (A), Koehler Type E (D), Algro Baress (E), Magno Satin (F).

Four of these substrates (Figure 9A,D–F) have been further examined, as they have a rather smooth and homogeneous surface and low, but non-zero, air permeance. The corresponding SEM images at a magnification factor of 1000 and 10,000 are shown in Figure 10 and Figure 11, respectively.

On the substrate p_e:smart paper type 2 (Figure 11A) the nanoporous coating, promoted by the producer, is clearly visible. The other substrates (Figure 11D–F) have typical mineral based coatings with flat platelets.

**Figure 12:**
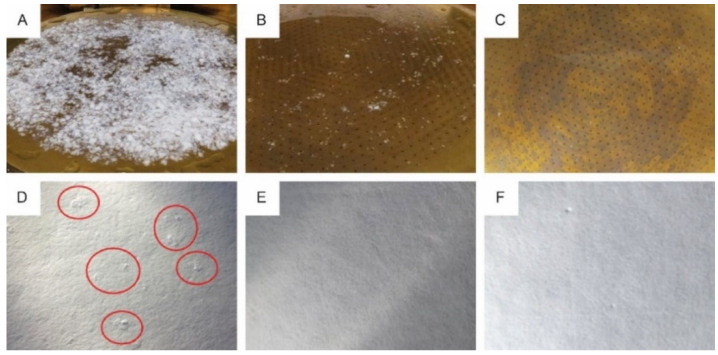
0.7 mm hole reject (**A**–**C**) and sheet adhesion test results (**D**–**F**) of p_e:smart paper type 2 (**A**,**D**), Algro Baress (**B**,**E**) and Magno Satin (**C**,**F**), respectively.

Based on the SEM analysis, three of the paper substrates (p_e:smart paper type 2 (A), Algro Baress (E) and Magno Satin (F) in Figure 11), which have quite different coatings, were selected to investigate their recyclability (without silver print). Figure 12 illustrates the results of the recyclability tests on both fiber-based substrates.

The reject of p_e:smart paper type 2, Algro Baress and Magno Satin amounted 56.0 m%, 0.7 m% and 0.8 m%, respectively. The reject of p_e:smart paper type 2 consisted of larger rigid pulp particles and non-paper coating particles (Figure 12A). In addition to the high amount of reject, the p_e:smart paper type 2 showed a few tacky particles still present in the hand sheets of the accept material (Figure 12D). In contrast, the Algro Baress and the Magno Satin showed no significant reject (Figure 12B,C) and no impurities were observed in the hand sheets (Figure 12E,F). Based on these recyclability tests, it was decided to continue working with the Magno Satin substrate in the next research steps.

### 3.2. Screen Printing of HF RFID Antennas

The antenna design (Figure 2) was screen printed with both conductive nano silver inks on the selected fiber-based substrate Magno Satin. It is a wood-free (i.e., removed lignin) graphic paper with a surface roughness and air permeance of 424 ± 24 nm and 0.2 ± 0.1 mL/min, respectively. For both ink-paper combinations, 10 different antennas were deposited, thermally cured and characterized more in depth.

First, a small deformation of the printed antennas, described as curling effect, was observed after the curing step at 150 °C. It indicates a moisture gradient across the substrate from the warmest zone to the coolest zone to finally facilitate evaporation at the coolest surface. The variation of moisture content causes local shrinkage of fibers in this cooler zone, creating the curling effect towards this ‘lower’ temperature region of the substrate [56]. Near infrared (NIR) curing, however, is described as a promising alternative to cure nano silver inks on different substrates without any deformation. This technique is less time-consuming, reduces the substrate temperature significantly and is even suitable for curing at high speed in roll-to-roll processes [57].

The basic electrical characteristics of the printed antennas (measured using a 2-probe method) differ significantly between both ink-paper combinations. The antennas printed with ‘Orgacon SI-P2000’ had an average series resistance and inductance of 38.2 ± 1.9 Ω and 2.35 ± 0.01 µH, respectively. According to earlier research, the inductance value is out of range to obtain functional antennas referring to the L6 design. In this case, a large amount of energy was captured by the antenna and for that reason, the antenna functioned as a filter for the requested data [54]. The screen printed antennas with ‘Loctite ECI 1011’ instead met the electrical requirements to obtain RFID functionality: the series resistance and inductance were 22.1 ± 2.3 Ω and 2.97 µH, respectively. The interaction of this ink-paper combination resulted in favorable electrical properties, ranging in the correct working frequency of the thin-film microchip; each tag’s code could be properly extracted with the reader device. Hence, the deposition of antenna design L6_4 with the ‘Loctite ECI 1011’ nano silver ink onto the Magno Satin substrate was concluded to be successful for screen printing HF RFID antennas on paper substrates. The reason for the functional antennas with the Loctite ink and the non-functional antennas with the Orgacon ink could be related to the viscosity and the corresponding line thickness. Due to the higher viscosity of the Orgacon ink at low shear rates, the ink tends to spread less after printing, resulting in deposited lines with higher thickness and in a higher resistivity value (as resistivity is sheet resistance multiplied by layer/line thickness). Different linewidths and thicknesses are achieved for both inks on the same paper, resulting, for this specific antenna design, in different resistance and inductance values. Achieving a working antenna with the Orgacon ink may also be possible by selecting a different antenna design and/or a different fiber-based substrate.

**Figure 13:**
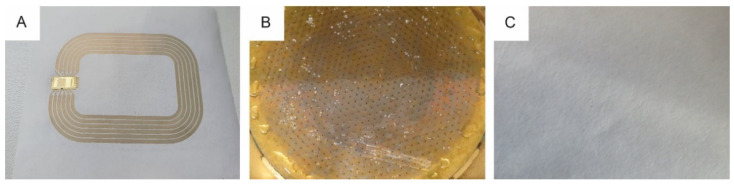
Screen printed RFID antenna with integrated IGZO-TFT microchip (**A**), 0.7 mm hole reject (**B**) and sheet adhesion test results (**C**) of functional screen printed RFID antennas with ‘Loctite ECI 1011’ ink and integrated microchips on Magno Satin substrate.

Next, the IGZO-TFT microchip was connected on top of the screen printed antenna with electrically conductive adhesive (Roartis, Belgium) by Quad Industries (Belgium) and the functionality of the HF RFID label was validated. An example of a screen printed antenna with integrated RFID chip is shown in Figure 13A.

Finally, the recyclability of the screen printed antennas with ‘Loctite ECI 1011’ ink on Magno Satin substrate with integrated RFID chips was investigated (Figure 13B,C). The reject amounted 1.4 m%, an increase of 0.6 m% compared to the base paper characterization. However, visual impurities of the accept were observed and for that reason, the printed antennas were assessed as “limited recyclable” according to the PTS-RH 021:2012 standard.

For all tested base substrates of the antennas, it applies that they will be recyclable when integrated into a cardboard packaging and get into the household paper and board collection. It is assumed that their mass share will be quite low compared to the overall substrate of the end product. If used as a mono-material, additional treatment steps should be considered. Separating the electronic components before recycling can also be beneficial to recuperate the used raw materials.

### 3.3. Design, Development and Validation of an RFID Assisted E-Fulfilment Packaging

The objective of this study was to develop a sustainable HF RFID assisted e-fulfilment packaging concept for multiple scanning interactions within third-party logistics as demonstrated in Figure 1. In brief, the customer purchases a product online via a web shop and the corresponding notification is received by the warehouse. They package the product in a foldable cardboard box with an integrated paper-based RFID label. The contents of the box, customer data, return data, etc. are included in a database that can be accessed via the RFID label and for that reason, the courier service can easily deliver the package to the correct destination (customer or central point). In a later stage, the empty cardboard box (with RFID label) is folded and returned to the warehouse as shown by the red-dotted line in Figure 1. Finally, the box’ status and RFID functionality are checked in the warehouse; an undamaged box is returned to the supply chain after deleting the previous RFID data whereas a damaged box is removed from the circulation and recycled. No labels need to be removed, nor adhered on top of each other for repeated use. A self-designed software tool (I4CRM, The Netherlands) supports this complete logistics cycle, and the package status can easily be switched from “ready to ship” to “shipped”, “shipped” to “ready to return” and finally “ready to reuse” [58]. Thanks to this software tool, the real-time location of the box is well-known in order to optimize the routing and scheduling to pick up the packages.

**Figure 14:**
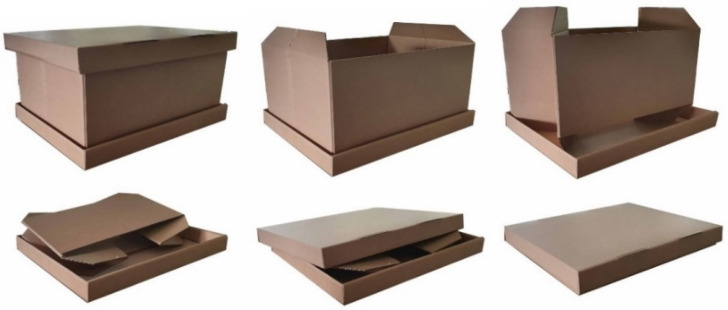
Folding principle of the developed tripartite cardboard package.

The following criteria were considered in the development of a box that can be reused as secondary packaging in logistics; (i) the design of the box must be user-friendly to motivate circularity, (ii) the use of adhesive labels and tape for closing must be avoided, as they can damage the packaging when teared off, and (iii) while using as little material as possible, a sufficient cardboard quality is required to withstand the logistical cycle several times [59,60]. The dimensions of the box (42.5 × 31.5 × 19.5 cm^3^) were based on frequently sold packages by logistical companies in Belgium. To make handling in the distribution cycle as efficient as possible, several foldable designs were studied, as this option saves space when the box is empty. In addition, the integration of the RFID label in the box will eliminate the need of informative labels. Foldable, clean boxes will motivate the customer to return the box to the central point. Taking innovation, compactness when folded, and usefulness for light and heavy weight products into account, prototypes of the tripartite foldable secondary packaging shown in Figure 14, were fabricated in collaboration with ROPAK (Genk, Belgium). The lid can be removed from the box and the trapezoidal flaps can be lifted to fold the box’ mantle. Finally, the lid recloses the folded package and is ready to return.

The selection of the cardboard type has a big impact on the durability, robustness and lifetime of reusable packages [61,62]. We started from 3-mm single wall cardboard to save costs and raw materials, and because of better foldability compared to double wall cardboard. However, preliminary studies showed that boxes of this type of cardboard are no option for multiple use in a logistics distribution cycle [59,60].

In this study, double-walled cardboard with an average single sheet thickness of 2.943 ± 0.008 mm was used. The impact energy to puncture this type of cardboard was 4.7 ± 0.2 J and it was not affected by either cross or machine direction. The edge crush resistance (ECT) of the cardboard was 630 ± 28 N.

Next, the theoretical compression strength of the box (BCT) was calculated using the formula of McKee (Equation (1)) [63,64].

(1) $$\mathrm{BCT}=k*\mathrm{ECT}*\;\sqrt{h*Z}$$

In this formula, k is a constant value of 5.874, ECT is the edgewise crush resistance (in N/cm), h is the cardboard thickness (cm) and Z refers to the box dimensions (perimeter) [2 × (H + L)] (cm). The calculated BCT value (2442 N) indicates the theoretical compressive strength of the cardboard box. However, the foldability of the package, which is required to implement the proposed concept, can significantly influence the real box’ strength. Indeed, when the box compression test was performed on the non-folded box with 2 strapping bands as closure system, a BCT value of 3226 ± 250 N was measured, which is significantly higher than the calculated theoretical value (2442 N). Both bottom and lid were not damaged, while the strapping bands prevented the mantle’s corners to pull outwards. Hence, it is notable that the folding lines did not affect the box’ compression strength.

As the final part of the validation, transport simulation experiments were carried out on the tripartite cardboard box sealed with 2 strapping bands. Table 2 presents the outcome of the different sequences of the transport simulation test cycle.

The box’ ability to withstand physical hazards occurring during manual handling (e.g., loading, sorting, palletizing…) was evaluated using the ASTM D4169 standard. No significant deformation was observed during both drop simulation tests. In addition, the loose-load vibration and vehicle vibration tests didn’t damage the cardboard package and no loss of loading occurred due to the strapping bands. However, the vehicle stacking experiment, did not meet the theoretical load L calculated from Equation (3) [65]:

(2) $$L=M_{f}*J*\frac{l*w*h}{K}*\frac{H-h}{h}*F$$

with M_f_ the shipping density factor (160 kg/m³), J the conversion factor (9.81 N/kg), l (w, h) the length (width, height) of the cardboard package (m), K (1 m³/m³), H the maximum height of stack in transit vehicle (2.7 m) and F a factor to account for the combined effect of the previous factors (7). The calculated load of 3685 N was higher than the measured load of 3226 ± 250 N and for that reason, the cardboard box was not completely in compliance with the ASTM D4169 standard. Despite this, a very rigid foldable cardboard package for e-fulfilment purposes was developed, considering the high safety factor number (7), a maximum stacking height of 2,7 m and a high recommended shipping density.

Finally, the paper label with the IGZO-TFT microchip connected on top of the screen printed antenna (3.2) was integrated into the cardboard package with transfer tape. A specific HF RFID reader was used to demodulate the microchip’s 12-bit data and send it to the home-built software application for this smart packaging using serial communication UART via a USB to TTL adaptor.

## 4. Conclusions

In this paper, 76 different (un)coated fiber-based substrates were studied regarding printability and ink compatibility for printed electronics. To select paper substrates to proceed with, the following threshold values were applied to the dataset; Van der Pauw sheet resistance below 50 mΩ/sq, no heat deformation at 150 °C, stylus profilometry surface roughness Ra below 1500 nm and Bendtsen air permeance below 40 mL/min. In addition, some mechanical and visual characteristics were considered to reduce the number of substrates. The Magno Satin substrate was finally used to print the antennas because it also performed the best in the recyclability test. This may not be true for other pieces of printed electronics, which require substrates with tailored surface properties such as p_e:smart. We realize that other paper substrates in the tested list can be useful in other applications.

The deposition of antenna design L6_4 with ‘Orgacon SI-P2000’on Magno Satin paper substrate showed an average series resistance and inductance of 38.2 ± 1.9 Ω and 2.35 ± 0.01 µH, respectively. These electrical characteristics did not allow to extract the RFID tags’ code of this ink-paper combination. Antennas screen printed with ‘Loctite ECI 1011’ met the electrical requirements to obtain RFID functionality, with series resistance and inductance of 22.1 ± 2.3 Ω and 2.97 µH, respectively. However, by adapting the paper substrate and/or the antenna design, working antennas might possibly be achieved as well for the Orgacon ink, as substrate, ink and antenna design can influence each other significantly. In addition, other printing technologies could be considered, such as aerosol jet printing for prototyping, offset, rotogravure or flexographic printing in combination with a suitable ink.

An IGZO-TFT microchip was connected on top of the screen printed antenna with electrically conductive adhesive and functional HF RFID labels were determined to be limited recyclable according the PTS-RH: 021/97 standard. Assuming that the mass share of the RFID label will be very low in the total paper and cardboard collection, no problems for recycling should be expected. Nevertheless, separating the RFID label may be a more sustainable solution to recuperate various raw materials.

The developed RFID label is ready for subsequent integration in a sustainable cardboard package for e-fulfilment purposes. A foldable design of the box was chosen to motivate its reuse in the logistical distribution cycle. Prototypes were validated in depth and transport simulation experiments showed that the cardboard packaging was, with exception of the vehicle stacking test, in compliance with the ASTM D419 standard.

Finally, a software tool was developed in order to register and to follow the real-time location of this smart packaging throughout the complete logistics cycle.

We conclude that this multidisciplinary approach to develop an easy-scalable screen printed HF RFID antenna on a low-cost paper substrate without an advanced coating layer and its subsequent integration in a smart box application for e-fulfilment purposes, is a good example for future implementation of hybrid electronics in sustainable smart packaging.

## Figures and Tables

**Table 1:** Ultimate selection of fiber-based substrates, compatible with printed electronics.

Paper Substrates *	Supplier	Ra (nm)	Air Permeance (mL/min)	Sheet Resistance (mΩ/sq)	
				Orgacon	Loctite
p_e:smart paper type 2	Felix Schoeller	114 ± 15	13 ± 1	24 ± 1	10 ± 1
Parade Label A	Sappi	315 ± 66	impermeable	21 ± 5	14 ± 1
Incada Exel	Iggesund Paperboard	351 ± 75	28 ± 1	15 ± 1	13 ± 3
Koehler TypE	Koehler Paper Group	360 ± 50	0.2 ± 0.1	25 ± 1	26 ± 1
Algro Baress	Sappi	404 ± 103	0.6 ± 0.1	22 ± 1	14 ± 1
Magno Satin	Sappi	424 ± 24	0.2 ± 0.1	28 ± 6	32 ± 2
Silvaboard	Smurfit Kappa	486 ± 43	20 ± 1	9 ± 1	14 ± 1
PG90	Grünperga	555 ± 93	impermeable	28 ± 1	19 ± 1
UPM Finesse Premium Silk H 90	UPM	623 ± 94	impermeable	11 ± 1	11 ± 1
Rochcoat	Rembrandt Verpakking	687 ± 155	32 ± 1	16 ± 1	13 ± 1
RHR21	DS Smith	898 ± 122	10 ± 2	27 ± 1	21 ± 1
Powercoat XD 125	Arjowiggins	1356 ± 76	5 ± 1	36 ± 1	34 ± 4
CoatedPAC LC WTCL140	DS Smith	1368 ± 191	8 ± 1	37 ± 1	24 ± 1

* All paper substrates were kindly provided by the industrial partners.

**Table 2:** Outcome of transport simulation test cycle of the tripartite cardboard box (ASTM D4169 DC13).

Simulation Experiment	Pass/Fail	Observations
A|Manual handling	Pass	Small, negligible compression damage of bottom corners
C|Vehicle stacking	Fail	Measured load (3226 N) ≠ calculated load (3685 N)
F|Loose-load vibration	Pass	No deformation of cardboard package
E|Vehicle vibration	Pass	No deformation of cardboard package
A|Manual handling	Pass	Small, negligible compression damage of top corner

## Data Availability

The data presented in this study are available in Supplementary Materials (Table S1).

## Supplementary Materials

The following are available online at https://www.mdpi.com/article/10.3390/ma14195500/s1. Table S1: PAPERONICS substrate selection based on basic weight, suppliers, surface roughness, air permeance and sheet resistance of 2 conductive silver inks.
