# Supplementary material for: Screen Printed Antennas on Fiber-Based Substrates for Sustainable HF RFID Assisted E-Fulfilment Smart Packaging
Source: Materials (Basel). 2021 Sep 23;14(19):5500. doi: 10.3390/ma14195500 (PMC8509514; doi:10.3390/ma14195500)
Supplement: Supplementary file 1 [file materials-14-05500-s001.zip › materials-1298708-supplementary.pdf]

Table S1. PAPERONICS substrate selection based on basic weight, suppliers, surface roughness, air permeance and sheet resistance of 2 conductive silver inks.

Description by supplier	Basic weight [g/m²]	Supplier	Surface Roughness [nm]	Surface Roughness [ml/min]	Air Permeance [ml/min]	Sheet Resistance [mΩ/sq]	
						Orgacon SI P-2000	Loctide ECI 1011
p_e:smart paper type 2	200	Schoeller Technocell	114 ± 15	2 ± 1	13 ± 1	24 ± 1	10 ± 1
p_e:smart paper type 3	170	Schoeller Technocell	169 ± 35	/	/	45 ± 7	30 ± 1
p_e:smart paper type 1	190	Schoeller Technocell	172 ± 23	0,2 ± 0,1	/	33 ± 1	25 ± 1
Unknown	295	Smurfit Kappa	279 ± 45	29 ± 7	50 ± 1	9 ± 1	13 ± 1
Powercoat HD 95	97	Arjowiggins	288 ± 70	10 ± 2	/	57 ± 1	71 ± 6
Parade Label A	120	Sappi	315 ± 66	11 ± 3	/	21 ± 5	14 ± 1
Incada Exel	280	Iggesund Paperboard	351 ± 75	14 ± 4	28 ± 1	15 ± 1	13 ± 3
Koehler TypE		Koehler Paper Group	360 ± 50	28 ± 18	0,2 ± 0,1	25 ± 1	26 ± 1
LumiSilk	130	Stora Enso	367 ± 51	11 ± 3	0,4 ± 0,1	13 ± 1	12 ± 1
Carta Solida	350	Smurfit Kappa	402 ± 67	46 ± 9	59 ± 7	9 ± 1	13 ± 1
Algro Baress	120	Sappi	404 ± 103	45 ± 17	0,6 ± 0,1	22 ± 1	14 ± 1
Powercoat HD 230	219	Arjowiggins	406 ± 77	9 ± 1	/	64 ± 3	54 ± 11
Magno Satin	135	Sappi (Papyrus)	424 ± 24	12 ± 3	0,2 ± 0,1	28 ± 6	32 ± 2
Silvaboard	300	Smurfit Kappa	486 ± 43	51 ± 15	20 ± 1	10 ± 1	14 ± 1
Korsnas Light	250	Rembrandt Verpakking	487 ± 63	51 ± 16	28 ± 2	17 ± 2	15 ± 1
Carta Elega	350	Smurfit Kappa	488 ± 34	64 ± 20	21 ± 2	10 ± 1	13 ± 1
Custom K	415	Rembrandt Verpakking	496 ± 4	86 ± 7	33 ± 4	19 ± 1	14 ± 2
PG90	90	Grünperga	555 ± 93	539 ± 384	/	28 ± 1	19 ± 1
CMPC Cartulina	270	Rembrandt Verpakking	558 ± 94	134 ± 57	28 ± 2	19 ± 3	13 ± 1
Accurate Top	290	Rembrandt Verpakking	564 ± 100	44 ± 13	20 ± 1	16 ± 1	12 ± 1
Metsaboard	340	Rembrandt Verpakking	572 ± 63	14 ± 2	60 ± 2	18 ± 1	13 ± 1
UPM Finesse Premium Silk H 90	90	UPM	623 ± 94	18 ± 2	/	11 ± 1	11 ± 1
Rochcoat	275	Rembrandt Verpakking	687 ± 155	39 ± 8	32 ± 1	16 ± 1	13 ± 1
BHW31	495	DS Smith	694 ± 131	2256 ± 86	24 ± 2	44 ± 6	21 ± 1
BGV035THT	445	Smurfit Kappa	706 ± 239	798 ± 26	22 ± 4	29 ± 2	20 ± 2
PG70	70	Grünperga	714 ± 212	136 ± 36	/	28 ± 2	20 ± 2
Koehler TypB		Koehler Paper Group	735 ± 57	31 ± 6	13 ± 1	29 ± 1	36 ± 7
Korsnas C	480	Rembrandt Verpakking	771 ± 113	54 ± 14	50 ± 2	21 ± 3	14 ± 1
UPM Finesse Premium Silk H 115	115	UPM	813 ± 29	20 ± 4	2 ± 1	12 ± 1	12 ± 1
RHR21	410	DS Smith	898 ± 122	335 ± 115	10 ± 2	27 ± 1	21 ± 1
PG60	60	Grünperga	900 ± 228	145 ± 14	/	29 ± 1	20 ± 1
Koehler TypA		Koehler Paper Group	945 ± 36	29 ± 4	/	42 ± 2	45 ± 3
Koehler TypD		Koehler Paper Group	1030 ± 79	53 ± 21	0,2 ± 0,1	35 ± 2	25 ± 3
MA8941	180	Ahlstrom Munksjö	1087 ± 122	70 ± 19	41 ± 2	35 ± 2	36 ± 4
CoatedPAC LC WTCL160	160	DS Smith	1093 ± 190	100 ± 19	17 ± 2	35 ± 2	25 ± 1
Powercoat XD 200	202	Arjowiggins	1319 ± 199	39 ± 5	31 ± 1	40 ± 2	39 ± 3
Powercoat XD 125	129	Arjowiggins	1356 ± 76	41 ± 5	5 ± 1	36 ± 1	34 ± 4
CoatedPAC LC WTCL140	140	DS Smith	1368 ± 191	109 ± 15	8 ± 1	37 ± 1	24 ± 1

Paper substrates indicated in color are given in Table 1. Ultimate selection of fiber-based substrates, compatible with printed electronics.

Table S1. PAPERONICS substrate selection based on basic weight, suppliers, surface roughness, air permeance and sheet resistance of 2 conductive silver inks.

Description by supplier	Basic weight [g/m²]	Supplier	Surface Roughness [nm]	Surface Roughness [ml/min]	Air Permeance [ml/min]	Sheet Resistance [mΩ/sq]	
						Orgacon SI P-2000	Loctide ECI 1011
Magazine Paper	52	Stora Enso	1887 ± 299	51 ± 2	161 ± 6	16 ± 2	13 ± 1
PackPro 7.5	45-60	PackPro	2103 ± 326	154 ± 55	/	13 ± 1	17 ± 1
UPM Digi Fine Jet	90	UPM	2250 ± 199	114 ± 15	618 ± 10	25 ± 2	39 ± 2
Tyvek 1073D, PE wit	75	DuPont	2257 ± 179	536 ± 126	655 ± 93	/	/
UPM Fine 140	140	UPM	2261 ± 69	136 ± 16	400 ± 11	21 ± 2	46 ± 1
R6310	400	Schutpapier	2303 ± 80	363 ± 55	83 ± 2	21 ± 3	16 ± 1
ML 9084	87	Mitsubishi	2335 ± 255	118 ± 4	507 ± 6	23 ± 6	18 ± 1
DNS Premium	120	Mondi	2393 ± 253	107 ± 13	717 ± 17	24 ± 1	48 ± 3
BergaMail+ 80	80	Stora Enso	2438 ± 425	174 ± 10	638 ± 3	15 ± 2	28 ± 3
Soporset Premium Offset	130	Navigator	2486 ± 331	220 ± 15	447 ± 15	19 ± 1	51 ± 6
EWV017THT	370	Smurfit Kappa	2571 ± 248	1493 ± 206	320 ± 11	24 ± 1	21 ± 2
UPM Poste 90	90	UPM	2575 ± 226	212 ± 21	625 ± 9	17 ± 1	26 ± 2
R2904 - alcohol barrier paper	100	Schutpapier	2627 ± 213	64 ± 12	0,6 ± 0,3	16 ± 4	13 ± 1
R6326	200	Schutpapier	2639 ± 342	339 ± 31	313 ± 26	15 ± 1	39 ± 6
RRE31	370	DS Smith	2745 ± 59	1525 ± 319	116 ± 6	28 ± 1	33 ± 1
UPM Poste 100	100	UPM	2883 ± 270	247 ± 156	827 ± 31	20 ± 3	61 ± 10
Acryl/Latex impregnated	130	Schutpapier	2884 ± 310	469 ± 80	23 ± 2	13 ± 1	12 ± 1
MultiOffset	120	Papyrus	2934 ± 552	362 ± 51	629 ± 14	48 ± 1	143 ± 20
CRE42	455	DS Smith	2973 ± 527	2818 ± 376	148 ± 2	37 ± 1	24 ± 1
BergaMail+ 90	90	Stora Enso	2980 ± 220	291 ± 40	445 ± 8	16 ± 1	20 ± 1
BergaMail+ 120	120	Stora Enso	2992 ± 283	288 ± 11	615 ± 19	19 ± 1	35 ± 4
Kraftpack	307	Rembrandt Verpakking	3070 ± 372	1373 ± 159	747 ± 38	21 ± 3	25 ± 1
UPM Poste 120	120	UPM	3134 ± 125	251 ± 18	829 ± 44	19 ± 2	59 ± 3
BergaMail+ 100	100	Stora Enso	3231 ± 467	237 ± 13	686 ± 17	17 ± 1	35 ± 2
UPM Fine 100	100	UPM	3356 ± 563	383 ± 39	695 ± 26	15 ± 1	22 ± 4
UPM Fine 90	90	UPM	3362 ± 570	340 ± 27	629 ± 10	16 ± 3	22 ± 3
UPM Poste 80	80	UPM	3528 ± 188	217 ± 18	674 ± 27	16 ± 1	46 ± 3
TL100	100	VPK Packaging	3528 ± 187	1045 ± 69	547 ± 22	20 ± 2	33 ± 2
REE32	405	DS Smith	3561 ± 528	2082 ± 136	226 ± 11	39 ± 1	89 ± 2
BVT055THT	460	Smurfit Kappa	3571 ± 525	1485 ± 123	159 ± 4	25 ± 1	22 ± 1
BKK089THT	715	Smurfit Kappa	3789 ± 459	1549 ± 163	207 ± 12	44 ± 1	88 ± 19
MA8067	147	Ahlstrom Munksjö	4226 ± 562	831 ± 8	Not measurable	137 ± 12	168 ± 12
CEE24	620	DS Smith	4338 ± 339	2305 ± 353	215 ± 8	34 ± 1	35 ± 2
FPG75	75	Grünperga	4395 ± 842	908 ± 137	2878 ± 78	92 ± 14	76 ± 4
BTT041BNL	385	Smurfit Kappa	4460 ± 1277	1557 ± 81	434 ± 16	27 ± 2	36 ± 5
C100	100	VPK Packaging	4692 ± 358	1327 ± 86	412 ± 19	19 ± 4	35 ± 3
M300	300	VPK Packaging	5301 ± 792	2011 ± 136	133 ± 6	24 ± 7	25 ± 1

Paper substrates indicated in color are given in Table 1. Ultimate selection of fiber-based substrates, compatible with printed electronics.
